# Dexamethasone to prevent kidney scarring in acute pyelonephritis: a randomized clinical trial

**DOI:** 10.1007/s00467-021-05398-w

**Published:** 2022-01-18

**Authors:** Neus Rius-Gordillo, Natàlia Ferré, Juan David González, Zaira Ibars, Ester Parada-Ricart, Maria Gloria Fraga, Sara Chocron, Manuel Samper, Carmen Vicente, Jordi Fuertes, Joaquín Escribano, on behalf of the DEXCAR Study Group

**Affiliations:** 1 Pediatrics Unit, Hospital Universitari Sant Joan de Reus, Reus, Spain; 2 Pediatric Nutrition and Human Development Research Unit, Universitat Rovira i Virgili, Reus, Spain; 3 Institut d’Investigació Sanitaria Pere Virgili, Tarragona, Spain; 4 Pediatrics Unit, Hospital General Universitario Santa Lucia, Cartagena, Spain; 5 Pediatrics Unit, Hospital Universitari Arnau de Vilanova, 25198 Lleida, Spain; 6 Pediatrics Unit, Hospital Universitari de Tarragona Joan XXIII, Tarragona, Spain; 7 Pediatrics Unit, Hospital de La Santa Creu i Sant Pau, Barcelona, Spain; 8 Pediatrics Unit, Hospital Universitari General Catalunya, Sant Cugat, Spain; 9 Pediatrics Unit, Pius Hospital de Valls, Valls, Spain; 10 Nephrology Department, Pediatrics Service, Hospital Clínico Universitario Virgen de La Arrixaca, Murcia, Spain; 11 Nuclear Medicine Service, Hospital Universitari Sant Joan de Reus, Reus, Spain; 12 Institut d’Investigació Sanitaria Pere Virgili, Sant Lloreç 21, 43201 Reus, Spain

**Keywords:** Acute pyelonephritis, Corticosteroids, Kidney scar, Children

## Abstract

**Background:**

Urinary tract infection (UTI) is one of the most common bacterial infections in childhood and is associated with long-term complications. We aimed to assess the effect of adjuvant dexamethasone treatment on reducing kidney scarring after acute pyelonephritis (APN) in children.

**Methods:**

Multicenter, prospective, double-blind, placebo-controlled, randomized clinical trial (RCT) where children from 1 month to 14 years of age with proven APN were randomly assigned to receive a 3-day course of either an intravenous corticosteroid (dexamethasone 0.30 mg per kg/day) twice daily or placebo. The late technetium 99 m-dimercaptosuric acid scintigraphy (> 6 months after acute episode) was performed to assess kidney scar persistence. Kidney scarring risk factors (vesicoureteral reflux, kidney congenital anomalies, or urinary tract dilatation) were also assessed.

**Results:**

Ninety-one participants completed the follow-up and were finally included (dexamethasone *n* = 49 and placebo *n* = 42). Both groups had similar baseline characteristics. Twenty participants showed persistent kidney scarring after > 6 months of follow-up without differences in incidence between groups (22% and 21% in the dexamethasone and placebo groups, *p* = 0.907). Renal damage severity in the early DMSA (β = 0.648, *p* = 0.023) and procalcitonin values (β = 0.065 *p* = 0.027) significantly modulated scar development. Vesicoureteral reflux grade showed a trend towards significance (β = 0.545, *p* = 0.054), but dexamethasone treatment showed no effect.

**Conclusion:**

Dexamethasone showed no effect on reducing the risk of scar formation in children with APN. Hence, there is no evidence for an adjuvant corticosteroid treatment recommendation in children with APN. However, the study was limited by not achieving the predicted sample size and the expected scar formation.

**Trial registration:**

Clinicaltrials.gov, NCT02034851. Registered in January 14, 2014.

**Graphical abstract:**

“A higher resolution version of the Graphical abstract is available as Supplementary information.”

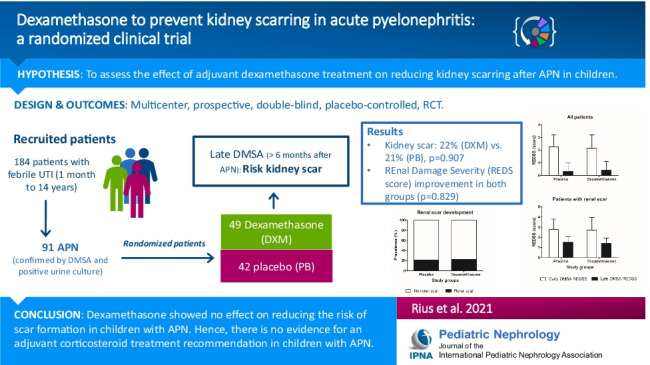

**Supplementary Information:**

The online version contains supplementary material available at 10.1007/s00467-021-05398-w.

## Introduction

Urinary tract infection (UTI) is one of the most common bacterial infections in childhood and is associated with long-term complications such as kidney scarring and later arterial hypertension, proteinuria, pre-eclampsia, and chronic kidney disease [1, 2].

Both age and sex modulate UTI incidence. It is estimated that 8–10% of girls and 2–3% of boys will suffer from a symptomatic urinary infection before the age of 7 [3–8]. Between 50 and 80% of patients with febrile UTI have acute pyelonephritis (APN) [9].

The pathogenesis of kidney scarring after APN is not completely understood. It is postulated that both acute kidney injury and scarring appear as a consequence of the inflammatory and immunological response triggered to eradicate kidney tissue infection through the activation of inflammatory mediators such as cytokines [10, 11]. The incidence of permanent kidney scarring after APN is highly variable (10–60%) [9, 12–14].

Recently, animal and human studies proposed a blockade of the inflammatory cascade involved in kidney scarring as a therapeutic option. In animal models, the use of corticosteroids, anti-inflammatory drugs (ibuprofen), dapsone, or melatonin/oxytocin resulted in a significant reduction in kidney scarring and its long-term consequences, probably due to an inhibitory effect on neutrophilic infiltration [15–19].

An important objective while treating patients with APN is to avoid permanent kidney damage. Therefore, any intervention that reduced kidney scarring would have a great impact, as it would decrease the incidence of chronic kidney failure or hypertension in adulthood. Previous results reported possible benefits of using corticosteroids that could lead to a change in the prognosis of APN patients [20–22]. Among corticosteroids, dexamethasone is an outstanding candidate due to its greater potency, extensive clinical knowledge, and previous benefits observed in the treatment of other infections, such as meningitis, where it reduced permanent sequelae [23].

The scarce literature published in pediatric APN patients shows differing methodologies and results [20–22]; thus, more trials are needed to establish well-based recommendations. Hence, our objective was to evaluate the effect of adjuvant dexamethasone treatment on kidney scarring after APN in children in a randomized clinical trial (RCT).

## Methods

This study was a phase III, double-blind, placebo-controlled, multicentric RCT performed in children with APN. The study was conducted in 8 Spanish hospitals corresponding to the acronym of DEXCAR.

### Study population

Children aged 1 month to 14 years with a first febrile (T > 38 °C) UTI episode who attended the emergency room service of the participating hospitals were considered for inclusion. Patients who fulfilled the criteria for hospital admission (as reported in the National Clinical Guidelines of Urinary Tract Infection [3]) were invited to participate in the study, and informed consent was obtained from caregivers. UTI was confirmed by a positive culture analysis in an appropriate urine collection method (clean catch in continent children (positive culture if > 100,000 CFU/mL) and catheterization in noncontinent children (> 10,000 CFU/mL)) [24]. All eligible children whose parents agreed to RCT enrollment underwent technetium 99 m-dimercaptosuric acid renal scintigraphy (DMSA) to confirm the presence of APN.

Exclusion criteria: endocrinological disease, immunosuppression treatment, cancer, or known uropathy (i.e., known VUR, CAKUT, and UTD). Patients with normal acute DMSA and those who developed a second UTI before the late DMSA assessment (minimum of 6 months of follow-up) were also excluded from the study and posterior analysis.

### Intervention

We performed randomization using EPIDAT 4.2 software (Consellería de Sanidade, Xunta de Galicia, España; Organización Panamericana de la salud (OPS-OMS); Universidad CES, Colombia). Concealing assignment was ensured by means of sealed envelopes that were prepared by an investigator not involved in the recruitment or analysis. We randomly assigned children in blocks of four in a 1:1 ratio to receive a 3-day course of either intravenous corticosteroid (dexamethasone 0.30 mg/kg, twice daily 3 days) or placebo (sodium chloride 0.9% with the same volume and administration regimen). Dexamethasone dose was similar to that used in other pediatric infectious diseases such as meningitis [23].

All patients received initial empirical antibiotic therapy according to each center protocol (first parenteral followed by oral therapy according to clinical criteria), and the adjustment depended on the antibiogram results.

### Follow-up and image assessment

Early DMSA was performed within the first 72 h of treatment. The criterion for APN was the presence of a multifocal or diffuse photopenia, with or without loss of kidney contours. A second DMSA was performed after a minimum of 6 months, and kidney scarring was defined as a persistent photopenic cortical defect(s) with or without loss of contour or volume compared with the first assessment examination. Images were acquired 3 h after radiotracer intravenous administration with a low energy general purpose (LEGP) parallel collimator and focused on the kidney area in anterior, posterior, and oblique projections [25]. A blinded unique nuclear medicine physician centralized DMSA assessment analyses to reduce interobserver variability. Renal function difference between both kidneys (in %, excluding bilateral impairment cases) was obtained from the DMSA assessment comparing radiotracer uptake. We used this difference % as a proxy indicator of functionality loss by the injury.

To assess the degree of kidney damage, we defined a score based on the affected areas of each kidney, according to criteria established for the Randomized Intervention for Children with Vesicoureteral Reflux (RIVUR) study, where each kidney is divided into 12 segments and the kidney damage is defined by 5-level grading system (briefly, grade 0 if no lesions are seen, grade 1 in case of 1–2 renal segments affected, grade 2 in case of 3–4 segments, grade 3 if more than 4 segments involved and grade 4 in those cases with global kidney atrophy) [26]. To assess the severity of damage of both kidneys, we defined a score as the arithmetic sum of the degree of involvement of each kidney. Thus, the renal damage severity score (REDSS) ranged from 0 points (no injuries on the DMSA) to an hypothetical 8-point score for a global diffuse photopenia of both kidneys. If the REDSS score was ≥ 3 points, we considered global damage as severe. The REDSS was calculated in both the early and the late DMSA scan evaluations.

During the acute phase, patients underwent abdominal ultrasound and voiding cystourethrography (VCUG). Abdominal ultrasound was performed by the local radiologist at each center, who was blinded to the treatment. The results were classified as a normal ultrasound, congenital anomalies of the kidney and urinary tract (CAKUT), or urinary tract dilatation (UTD) according to the last multidisciplinary consensus UTD classification system [27, 28]. Voiding cystourethrography was performed 2–4 weeks after APN diagnosis. Vesicoureteral reflux (VUR) was graded I–V according to the US International Study Committee on Vesicoureteral Reflux in Children [29]. VUR was defined as nondilated VUR (grades 1 and 2) and dilated VUR (grades 3–5).

### Other clinical and analytical data

Fever (ºC) was measured at the emergency room service, and days of previous fever (> 38ºC) were recorded as reported by parents. We performed a blood and urine test at the time of admission. We analyzed the hemogram, C-reactive protein (CRP), procalcitonin (PCT), ionogram, urea, and creatinine. Blood and urine cultures were performed in all patients before the initiation of antibiotherapy.

### Statistical analysis

Quantitative variables were expressed as the means (± SD) or medians (interquartile ranges) after assessing the normal distribution of variables with a Kolmogorov–Smirnov test. Categorical variables were expressed as n and percentage (%). T-tests or Mann–Whitney U-tests were used for statistical comparisons during the cross-sectional analysis between groups. Pearson’s chi-squared test was used for the statistical comparison of the categorical data. Correlations between quantitative variables were performed by Pearson or Spearman test as appropriate. We performed logistic regression analysis to assess the effect of the intervention on later kidney scar presence, adjusting for relevant confounders (vesicoureteral reflux, germ, age, sex, etc.).

The statistical significance was accepted at *p* < 0.05. The data management and analyses were conducted using IBM SPSS Statistics software version 27.0 (IBM Corp., Armonk, NY, USA).

The sample size (81 per group) was calculated using data reported by Huang et al. [20] that reported a 50% reduction in kidney scarring risk among infants treated with corticosteroids and assuming a kidney scarring prevalence in the placebo group of about 40%. We assumed an attrition rate during follow-up of 10%, an α error of 0.05, and a power of 80%. We used the Epidat 4.2 program for these calculations (see above).

### Legal and ethical considerations

The study fulfilled the principles of the Declaration of Helsinki and was approved by the local ethics committees of each participating center as well as by the Spanish Regulatory Agency (Agencia Española del Medicamento y Productos Sanitarios). Written informed consent was obtained from all parents or caregivers. The study followed the recommendations of the CONSORT guidelines [30]. This clinical trial was registered at Clinicaltrials.gov as NCT02034851.

## Results

**Fig. 1:**
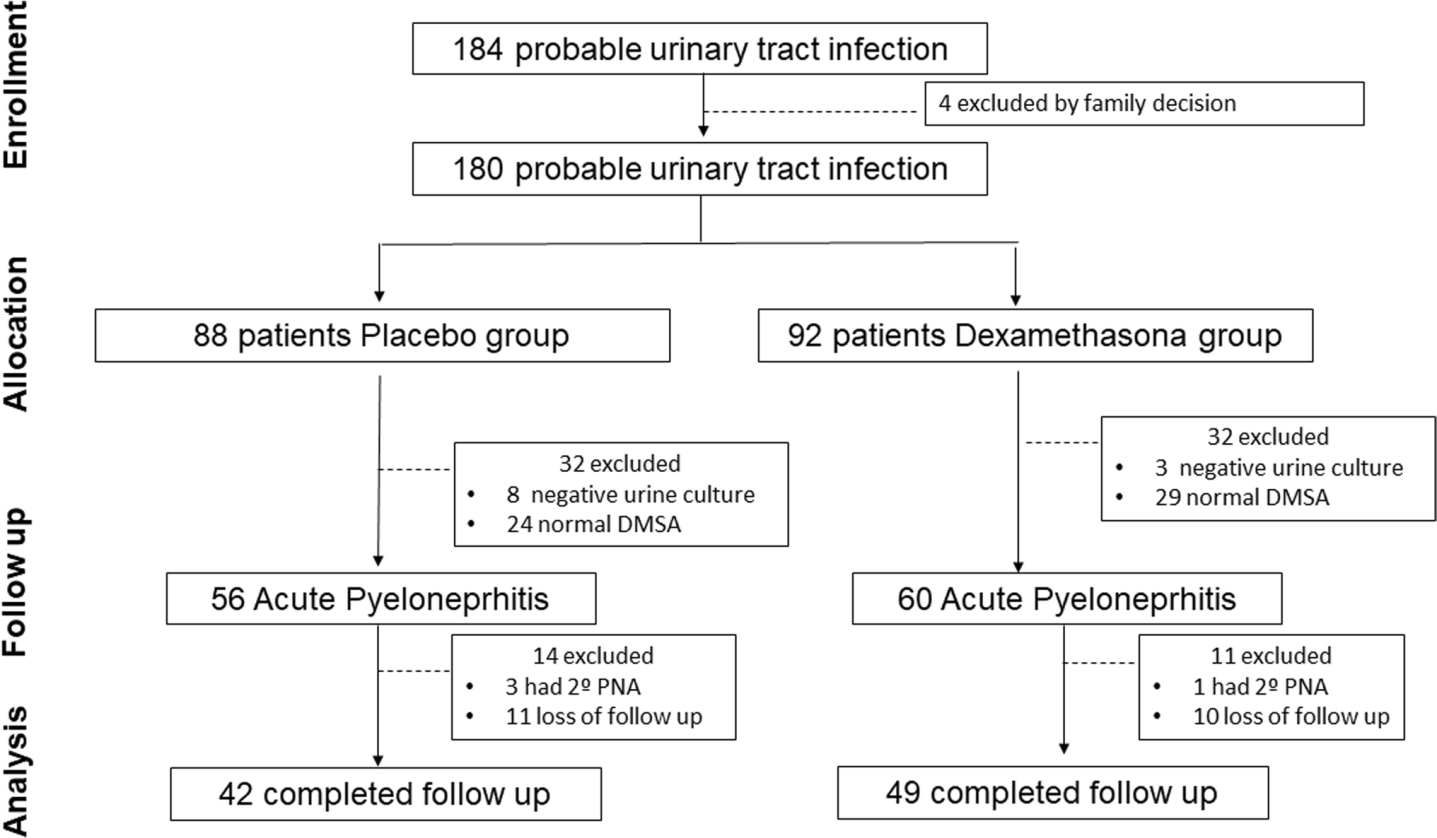
Flow chart

One hundred eighty-four patients were recruited between April 2013 and April 2018. After recruitment, 4 parents withdrew their consent; therefore, 180 infants were randomly allocated to receive dexamethasone (*n* = 92) or placebo (*n* = 88). Acute pyelonephritis was confirmed by DMSA and a positive urine culture in 116 infants (60 in the dexamethasone group and 56 in the placebo group). Ninety-one patients (49 and 42 in the dexamethasone and placebo groups, respectively) completed the follow-up and were finally analyzed. Figure 1 shows the flowchart of the study. Twenty-one infants were lost to follow-up (11 in the placebo group and 10 in the intervention one) and four had a second APN before the late DMSA examination (3 in the placebo and 1 in the intervention group). There were no differences between study groups in the number or the reasons for withdrawing (including UTI recurrence; 5.4% vs. 1.7% in placebo and intervention groups respectively, *p* = 0.5623).

Among the included 91 infants, 74% were females. The median age was 9 months (IQR 4–21 months, from 1 month to 11 years). Eighty percent of infants (*n* = 73) were younger than 2 years, and almost 63% (*n* = 57) were younger than 1 year. As expected, 98% of infections were caused by *Escherichia coli*. The empiric antibiotic therapy administered was gentamicin in 43% of patients (n = 39), amoxicillin-clavulanic acid in 29% (*n* = 26) and cephalosporin in 29% (*n* = 26). None of the patients had associated bacteremia or other complications including infection by resistant microorganisms. Abdominal ultrasound was abnormal in 11 patients (12%) (9 UTDs and 2 CAKUTs (double ureteral system in one patient and renal ectopy in the other)). Among patients with any UTD, none of them were classified in the high-risk group according to the new consensus classification (44% low risk and 56% moderate) [28].

Vesicoureteral reflux was confirmed in 12 patients (15% among the 83 who completed the VCUG assessment). All patients showed a coincidence of the APN with the reflux side. Reflux severity was classified as dilated (grades 3–5) in 4 patients.

Table 1 shows the patient characteristics by intervention group. Both groups were comparable in terms of age, sex distribution, fever before admission, basal blood analysis variables, germ, renal uropathy presence (VUR, CAKUT or UTD), and early REDSS. Although both study groups showed a similar % of patients with VUR, the number of patients classified as having dilated reflux (grades 3–5) tended to be higher in the dexamethasone group, without reaching statistical significance. Both groups were also comparable in terms of time delay between diagnosis and the beginning of study product administration (9.2 ± 7.8 vs. 8.5 ± 8.2 h for the dexamethasone and placebo groups, *p* = 0.531).

**Table 1:** Patients’ basal characteristics by treatment group

Variable	Placebo (*n* = 42)	Dexamethasone (*n* = 49)	*P* value
Age (months; median, IQR)	9.5 (4.0–17.3)	8.0 (4.0–21.5)	0.808
Sex (n; % of females)	30; 72%	37; 76%	0.660
Fever before hospital admission (days)	3.4 ± 3.5	2.2 ± 2.3	0.168
Biochemical variables:			
* Leucocytes* (mm^3^)	20.0 10^3^ ± 6.9 10^3^	20.7 10^3^ ± 6.3 10^3^	0.657
* Neutrophils* (mm^3^)	12.2 10^3^ ± 5.7 10^3^	12.3 10^3^ ± 5.0 10^3^	0.839
* CRP* (mg/dL)	13.5 ± 9.7	11.3 ± 5.9	0.608
* PCT* (ng/mL)	10.4 ± 17.8	5.6 ± 8.1	0.321
Germ (n; % of *E. coli*)	42; 100%	47; 96%	0.544
Uropathy:			
* CAKUT* (n; %)	1; 2%	1; 2%	0.544
* Urinary tract dilatation* (n; %)	5; 12%	4; 8.2%	0.551
VUR (n; %)			
* Any VUR* (n; %)	5; 12%	7; 14%	0.738
* Dilated VUR (grades 3–5)* (n; %)	1; 20%	3; 43%	0.400
Early REDSS (score)	2.3 ± 1.0	2.2 ± 1.1	0.673

*CRP* C-reactive protein, *PCT* procalcitonin, *CAKUT* congenital anomalies of kidney and urinary tract, *UTD* urinary tract dilatation, *VUR* vesicoureteral reflux, *Early REDSS* renal damage severity score at the early DMSA. The results are presented as the means ± SD, median ± IQR (interquartile range: 25 centile–75 centile) or n, % as appropriate

Early REDSS was directly correlated with differences in renal function between kidneys (*r* = 0.287, *p* = 0.008), showing an agreement of this score with functional loss in the affected kidney (analysis performed in the unilateral affected patients). REDSS also correlated with acute phase reactants (PCR: *r* = 0.320, *p* = 0.006 and PCT: *r* = 0.496, *p* < 0.001).

Overall, a kidney scar was observed in 20 patients (22% of included patients: 1 grade 4, 9 grade 2, and 10 grade 1). Kidney scarring was observed in 16% of infants younger than 1 year, 21% in those younger than 2, and 28% among children older than 2, but these differences were not statistically significant (*p* = 0.358). Table 2 shows the clinical and biochemical characteristics of patients according to kidney scar presence. Infants who developed kidney scarring showed a higher early REDSS compared to those who did not develop scarring (2.7 ± 1.1 vs. 2.1 ± 0.9 points in the scar present and absent groups, *p* = 0.030). There were no differences in any of the other studied variables (Table 2). Patients with VUR showed a threefold higher risk of developing a kidney scar, without reaching statistical significance (OR 3.19 (0.87–11.64, *p* = 0.08)).

**Table 2:** Study patients characteristics by kidney scar development

Variable	Kidney scar (*n* = 20)	No kidney scar (*n* = 71)	*P* value
Age (months; median, IQR)	13.4 (4.0–33.8)	8.0 (4.0–21.0)	0.334
Sex (n; % of females)	16; 80%	51; 72%	0.656
Fever:			
* Maximum temperature (°C)*	38.9 ± 1.0	38.7 ± 1.0	0.260
* Fever before clinics admission (days)*	2.8 ± 2.7	2.8 ± 3.1	0.896
* Fever after clinics admission (days)*	1.4 ± 1.2	1.2 ± 0.8	0.418
Laboratory results:			
* Leucocytes* (mm^3^)	18.8 10^3^ ± 5.4 10^3^	20.8 10^3^ ± 6.8 10^3^	0.227
* Neutrophils* (mm^3^)	11.7 10^3^ ± 4.6 10^3^	12.4 10^3^ ± 5.5 10^3^	0.543
* CRP* (mg/dL)	144.5 ± 80.0	117.4 ± 78.5	0.113
* PCT* (ng/mL)	20.0 ± 27.1	5.3 ± 6.6	0.230
Germ (n; % of *E. coli*)	19; 95%	70; 99%	0.917
Uropathy presence:			
* CAKUT* (n; %)	1; 5%	1; 1%	0.917
* Dilatation* (m; %)	3; 15%	6; 8.4%	0.386
VUR (n; %)	5; 28%	7; 11%	0.069
Early REDSS (score)	2.7 ± 1.1	2.1 ± 0.9	0.030

*CRP* C-reactive protein, *PCT* procalcitonin, *CAKUT* congenital anomalies of kidney and urinary tract, *UTD* urinary tract dilatation, *VUR* vesicoureteral reflux, *Early REDSS* renal damage severity score at the early DMSA. The results were presented as means ± SD, median ± IQR (interquartile range: 25 centile–75 centile) or n, % as appropriate

**Fig. 2:**
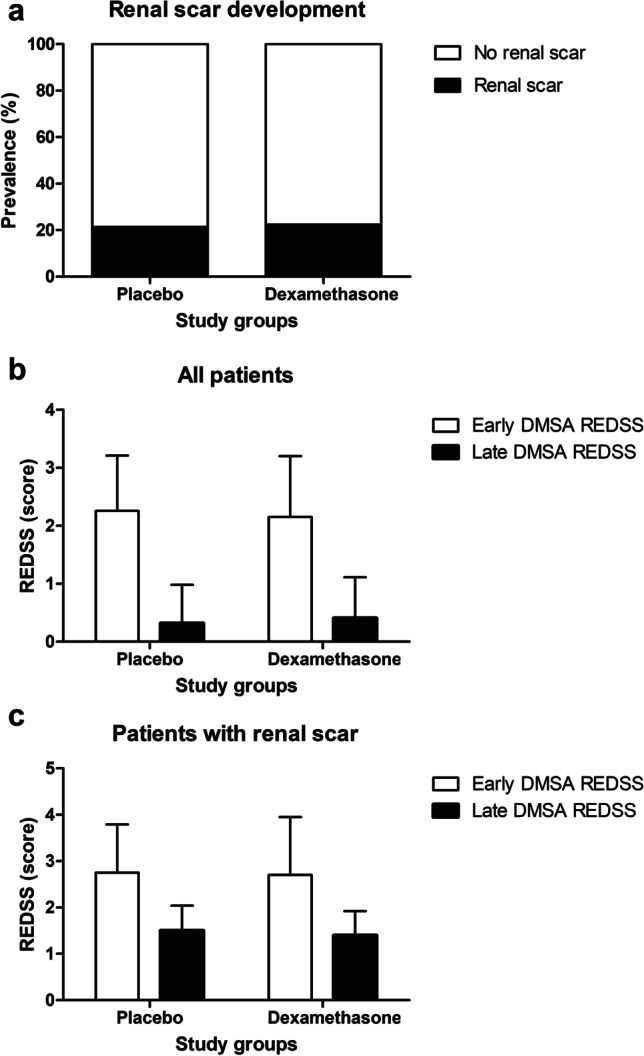
Kidney scar development and severity according to treatment group

The effect of the intervention on kidney scar development and severity is shown in Fig. 2. The incidence of kidney scarring was similar in both groups (22% and 21% in the dexamethasone and placebo groups, respectively, *p* = 0.907) (Fig. 2a). Treatment with dexamethasone did not enhance remission in kidney damage, nor did it enhance remission in all children (Fig. 2b) or in those with persistent kidney damage (Fig. 2c). The REDSS (both in the early and late phase) were similar between groups (early: 2.15 ± 1.05 vs. 2.26 ± 0.95 in the dexamethasone and placebo groups, p = 0.673; late: 0.41 ± 0.70 vs. 0.32 ± 0.66 in the dexamethasone and placebo groups, p = 0.848) (Fig. 2b). The kidney damage (REDSS) improved in most patients (94%) irrespective of the study group. Moreover, among those children with permanent scarring, the magnitude of the improvement was also independent of the treatment, as both dexamethasone and placebo groups showed a 42% reduction of REDSS (*p* = 0.988) (Fig. 2c). The dexamethasone group showed a significant reduction in fever days after clinic admission (0.9 ± 0.8 days vs. 1.7 ± 0.9 days, *p* < 0.001), but the maximal temperature reached was not different between the study groups.

We analyzed the possible effect of the treatment in those infants belonging to higher risk subgroups (i.e., age, diagnostic delay (measured as days of fever before admission), elevated acute phase reactants, UTD, or VUR) (Table 3). The incidence of kidney scarring was similar among the intervention groups in all the specific risk factors studied. These results were confirmed by logistic regression that assessed the effect of treatment adjusted by possible confounders***.*** Kidney scar development was significantly modulated by early REDSS (β = 0.648, *p* = 0.023) and PCT values (β = 0.065 *p* = 0.027), whereas VUR grade showed a tendency to modulate kidney scar development (β = 0.545, *p* = 0.054). None of the other variables showed a significant relationship with kidney scar development, irrespective of the study treatment groups or adjustment by confounders.

**Table 3:** Effect of intervention on kidney scar development in higher risk-specific groups

Risk factor	Dexamethasone *(n; % kidney scar)*	Placebo *(n; % kidney scar)*	Odds ratio Odds (95%CI)	*P* value
Age				
* General sample*	11; 22%	9; 21%	1.06 (0.39, 2.88)	0.907
* Older than 1 y*	5; 26%	6; 40%	0.54 (0.13, 2.29)	0.400
* Older than 2 y*	2; 20%	3; 38%	0.42 (0.05, 3.43)	0.416
Diagnostic delay				
≥ *2 days of fever before treatment*	3; 12%	6; 25%	0.41 (0.09, 1.87)	0.249
≥ *3 days of fever before treatment*	3; 18%	9; 32%	0.46 (0.09, 2.25)	0.341
Acute phase reactants				
* CRP* ≥ *5 mg/dL*	10; 23%	8; 23%	0.99 (0.34, 2.86)	0.989
* CRP* ≥ *8 mg/dL*	9; 27%	7; 25%	1.12 (0.35, 3.55)	0.841
* PCT* ≥ *0.5 ng/mL*	3; 13%	5; 20%	0.57 (0.12, 2.71)	0.481
* PCT* ≥ *2 ng/mL*	3; 23%	4; 25%	0.90 (0.16, 5.00)	0.904
VUR	4; 57%	1; 20%	5.33 (0.37, 75.77)	0.216
UTD ultrasound	2; 50%	1; 20%	4.00 (0.21,75.66)	0.813
Severe early DMSA damage (REDSS ≥ 3)	4; 40%	5; 36%	1.20 (0.23, 6.39)	0.831

*CRP* C-reactive protein, *PCT* procalcitonin, *VUR* vesicoureteral reflux, *UTD* urinary tract dilatation, *REDSS* renal damage severity score

## Discussion

Acute pyelonephritis can cause kidney scars and permanent kidney damage [2]. In our study, the presence of kidney scarring was observed in 20 patients (22%), with a slight tendency to a higher risk of scarring in patients older than 2 years or those with the presence of VUR, as previously observed [6, 13, 14, 22, 31]. Children with more extensive parenchymal involvement at admission had an increased risk of scarring (REDSS 2.7 ± 1.1 vs. 2.1 ± 0.9, p = 0.030).

Previous animal studies provided evidence for the efficacy of corticosteroids in preventing kidney scarring that was attributed to a decrease in inflammatory cytokines [15, 16]. Accordingly, a RCT performed in children with APN verified by DMSA demonstrated a protective effect of oral corticosteroids on scar development [20]. That RCT, which included patients with severe damage to the kidney parenchyma in the acute phase and a high risk of scarring, achieved an almost 50% reduction in the number of scars in the prednisolone group, which included only 18 infants. The number of children with scars was very high, since even in the group treated with corticosteroids it was 33%, clearly exceeding the 22% of children with scars in our trial. Our study, which included a larger number of patients with different degrees of parenchymal impairment, failed to demonstrate a protective effect of iv. dexamethasone treatment on kidney scarring. Other trials have investigated the effect of different doses of dexamethasone (both via oral or intravenous administration) on children with febrile UTI and failed to demonstrate a protective effect of corticosteroid administration (although they showed a trend towards a risk reduction) [21, 22]. The study by Shaikh et al. [22] had some important limitations, as they did not confirm APN by DMSA in the acute phase. Thus, the results could be masked by a different proportion of patients with APN, hiding the interpretation of the intervention. Furthermore, as the authors point out, they did not reach the desired sample size to prevent a loss of statistical power. It is true that in clinical practice, it is not common to perform a DMSA in the acute phase, but it does not allow us to draw completely valid pathophysiological conclusions. Finally, it is also worth mentioning that dexamethasone treatment was oral in Shaikh et al., and compliance was defined as 80% of doses administered as reported by parents (which does not exclude a misreporting effect in comparison with those trials performed with iv. treatment under hospitalization). Another trial, the study by Ghaffari et al. [21], shows important methodological inaccuracies and mainly aimed to demonstrate the relationship between permanent high levels of IL-6 and IL-8 during the acute phase of febrile UTI and the subsequent presence of kidney scars, irrespective of the received treatment. Actually, this study did not show a statistically significant protective effect of dexamethasone on kidney scarring, and if we analyze only the patients with APN demonstrated by DMSA, dexamethasone had the same effect in both groups (33% kidney scars); therefore, the data of Ghaffari et al. are very similar to our results.

Different prognostic factors have been postulated to increase the risk of kidney scarring after pyelonephritis [6, 13, 14, 32–37]. We analyzed whether treatment with dexamethasone reduced the risk of scarring in specific high-risk patients, and the obtained ORs were not significant for these subgroups (i.e., age older 2 years, prolonged fever, VUR, elevated CRP, or magnitude of kidney damage in DMSA). The lack of signification could be affected by a loss of statistical power due to the limited sample size, especially in the subgroup analyses. The analysis of the magnitude of kidney damage allowed us to assess whether our results reproduced the protective effects observed by Huang et al. [20] in severe impairment-affected patients. In our patients, corticosteroids showed the same effect as placebo, reducing parenchymal damage and kidney scarring in both patients with mild and severe impairment (Fig. 2). It is also noteworthy that in infants with VUR, who could be considered the group with the highest risk of scarring, no effect could be demonstrated, possibly due to the small number of patients with dilated reflux in each intervention group.

Finally, we analyzed whether these factors could influence kidney scarring development regardless of corticosteroid treatment. We found that the presence of VUR and a higher early REDSS tended to be associated with kidney scarring, as both variables were increased among infants with kidney scarring (with a trend towards statistical significance, Table 2). These results were confirmed by logistic regression, as both variables modulated the presence of permanent scarring (p = 0.023 for REDSS at the early DMSA and p = 0.054 for VUR). These data confirm previous findings described in the literature and allow us to conclude that the results are in line with those described previously [6, 13, 33].

One of the limitations of our study is that we did not reach the goal of 80 included children per group because a high number of enrolled patients were excluded due to normal acute phase DMSA, despite meeting the clinical criteria for APN. Moreover, the percentage of kidney scarring in our study was 22%, which was substantially lower than that we had foreseen during the sample size calculation (40%). Thus, our study sample was probably underpowered compared to other trials, because we excluded children with already known specific factors to avoid any artifact from previous scars. Maybe the low proportion of non-*E. coli* infections and the relatively low number of patients with CAKUT or VUR that are associated with a higher chance of scarring could have influenced our results. So, the study was underpowered to detect a difference, but it is still relatively likely that our conclusions are true, as there was no tendency towards a difference between the groups. To reduce interobserver discrepancies between centers, we centralized the assessment of DMSA images to one nuclear medicine physician, resulting in a loss of some data. However, despite these limitations, ours is the study with the largest number of patients included in both RCT arms currently published among those which have investigated the effect of corticosteroid treatment on kidney scarring after APN in children.

In summary, our study shows that dexamethasone plus antibiotics did not reduce the risk of scar formation in children with APN compared to antibiotic therapy alone. As other authors have shown, acute severity damage (evaluated in our study as early REDSS), PCT values, and the presence of VUR were independent risk factors for scar formation.

## Supplementary Information

Below is the link to the electronic supplementary material.

Supplementary file1 (PPTX 143 KB)

## Data Availability

The datasets generated during and/or analyzed during the current study are available from the corresponding author on reasonable request.
