# Supplementary material for: Dexamethasone to prevent kidney scarring in acute pyelonephritis: a randomized clinical trial
Source: Pediatr Nephrol. 2022 Jan 18;37(9):2109–18. doi: 10.1007/s00467-021-05398-w (PMC9307518; doi:10.1007/s00467-021-05398-w)
Supplement: Supplementary file 1 — Supplementary file1 (PPTX 143 KB) [file 467_2021_5398_MOESM1_ESM.pptx]

## Slide 1
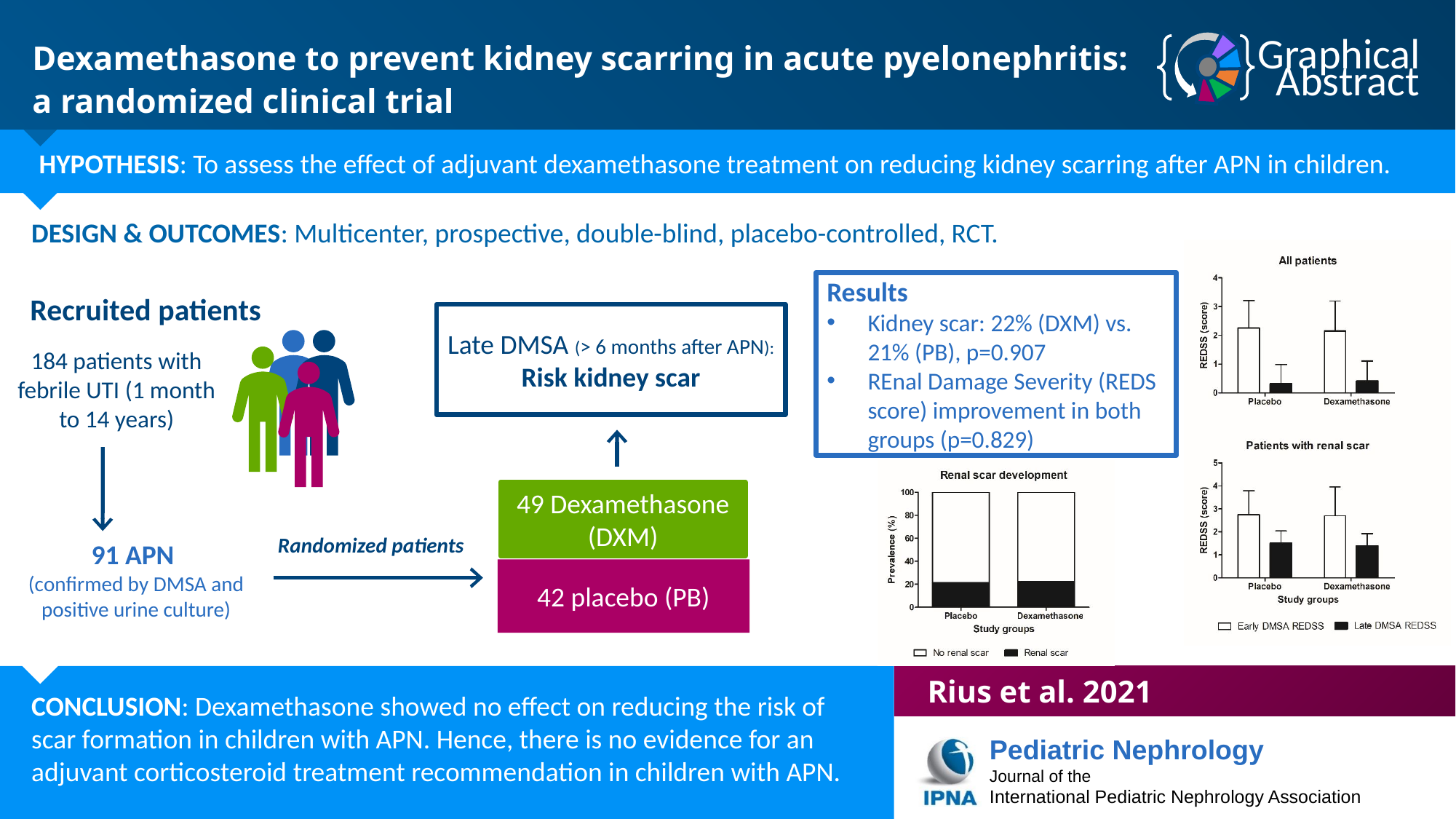

Dexamethasone to prevent kidney scarring in acute pyelonephritis:
a randomized clinical trial
HYPOTHESIS: To assess the effect of adjuvant dexamethasone treatment on reducing kidney scarring after APN in children.
DESIGN & OUTCOMES: Multicenter, prospective, double-blind, placebo-controlled, RCT.
Results
Kidney scar: 22% (DXM) vs. 21% (PB), p=0.907
REnal Damage Severity (REDS score) improvement in both groups (p=0.829)
Recruited patients
Late DMSA (> 6 months after APN): Risk kidney scar
184 patients with febrile UTI (1 month to 14 years)
49 Dexamethasone (DXM)
Randomized patients
91 APN
(confirmed by DMSA and positive urine culture)
42 placebo (PB)
Rius et al. 2021
CONCLUSION: Dexamethasone showed no effect on reducing the risk of scar formation in children with APN. Hence, there is no evidence for an adjuvant corticosteroid treatment recommendation in children with APN.
